# Recent trends in cosmic radiation exposure onboard aircraft: effects of the COVID-19 pandemic on Japanese in-flight doses

**DOI:** 10.3389/fpubh.2025.1554332

**Published:** 2025-04-17

**Authors:** Hiroshi Yasuda, Hiroto Motoyama, Kazuaki Yajima

**Affiliations:** ^1^Research Institute for Radiation Biology and Medicine (RIRBM), Hiroshima University, Hiroshima, Japan; ^2^School of Medicine, Hiroshima University, Hiroshima, Japan; ^3^National Institutes for Quantum Science and Technology, Chiba, Japan

**Keywords:** cosmic radiation, commercial flight, aviation dose, Japanese, per-capita, COVID-19 pandemic

## Abstract

**Background:**

Galactic cosmic radiation (GCR) is a naturally occurring environmental radiation that originates from outer space. GCR is modulated by solar activity, and its intensity increases with increasing geomagnetic latitude and altitude, reaching a peak of up to approximately 20 km in the atmosphere. Therefore, commercial flight passengers (flyers) are exposed to elevated levels of cosmic radiation while flying onboard commercial aircraft. Although the recent COVID-19 pandemic, which began in early 2020, is believed to have significantly affected public exposure to cosmic radiation, this impact is yet to be quantified.

**Methods:**

Based on the official records of Japanese flyers, their annual per-capita doses (APCDs) of cosmic radiation exposure on international and domestic flights were calculated using the established code JISCARD EX over a 7-year period from 2014 to 2020 (including the first year of the pandemic). For estimating the APCDs on international flights, the world was divided into eight regions. The aviation route dose to a representative city in each region was determined at three cruising altitudes: 34,000 ft. (10.4 km), 37,000 ft. (11.3 km), and 40,000 ft. (12.2 km).

**Results:**

At a typical cruising altitude of 37,000 ft., the flyer-average APCD from international flights was estimated to be approximately 60 μSv y^−1^, while the APCD from domestic flights was approximately 2 μSv y^−1^ over the target period, including the pandemic year (2020). These results indicate that the distribution of Japanese travel destinations did not change significantly during the pandemic period. In contrast, the population-average APCD significantly decreased from approximately 10 μSv y^−1^ in the pre-pandemic period (2014–2019) to 2 μSv y^−1^ in 2020, representing a reduction of more than 80%, which corresponds to a decline in the number of travelers.

**Conclusion:**

The results of this study indicate that the population-average APCD of Japanese flyers decreased significantly during the COVID-19 pandemic, while the flyer-average APCD remained largely unchanged. Further studies will be performed to determine APCDs for the subsequent period and to assess the overall effect of the pandemic on public health.

## Introduction

During travel using commercial aircraft, flight passengers (flyers) and aircraft crews are exposed to elevated levels of galactic cosmic radiation (GCR) originating from outer space. Cosmic radiation is one of the physical health factors impacting passengers during flights, along with vibration, high noise, low humidity, decreased oxygen levels, and intense electromagnetic fields. Flyers are also exposed to various chemical, biological, and psychosocial stressors that can affect multiple functional systems and cause specific diseases (1). Epidemiological studies have indicated that the risk of certain cancers, such as skin cancer and breast cancer, may be higher in aircraft crews, although a causal relationship is yet to be established (2–4).

The effective dose rate at typical aviation altitudes (10–12 km) is approximately 100 times higher than that at the ground level, reaching up to 7 μSv h^−1^ during flights at higher latitudes (5). This elevated natural radiation exposure in aviation has been acknowledged by the International Commission on Radiological Protection (ICRP), which recommended that the exposure of personnel to cosmic radiation during the operation of commercial jet aircraft should be treated as occupational (6, 7). In line with these recommendations and corresponding legal frameworks, the exposure of aircraft crews to natural radiation has been managed at the country or region level, as observed in the United States (8), Europe (9), and Japan (10).

While some information on cosmic radiation exposure among aircraft crews has been reported (10–13), data on the cosmic radiation exposure of the public onboard civil aircraft remains limited (14, 15). Although it is believed that their doses are not as high as those of aircraft crews, except for a small group of frequent flyers, it is important to clarify the overall situation of radiation exposure and the associated health risks to the public in daily life. In particular, the recent occurrence of the COVID-19 pandemic in 2020 has significantly impacted the lives of many people and dramatically altered their exposure to various environmental stressors (16, 17). Fear of serious consequences of infection with severe acute respiratory syndrome coronavirus 2 (SARS-CoV-2) and international and domestic travel restrictions and quarantine regulations have resulted in a significant drop in the number of flyers worldwide (18, 19). Although this decline is expected to have reduced the cosmic radiation exposure of travelers, the extent of the reduction remains unquantified. The purpose of this study was to provide quantitative information on the changes in in-flight cosmic radiation doses for flyers due to the COVID-19 pandemic, with the aim of contributing to ongoing discussions about the overall impact of the pandemic on public health.

## Methods

### Target period

**Figure 1:**
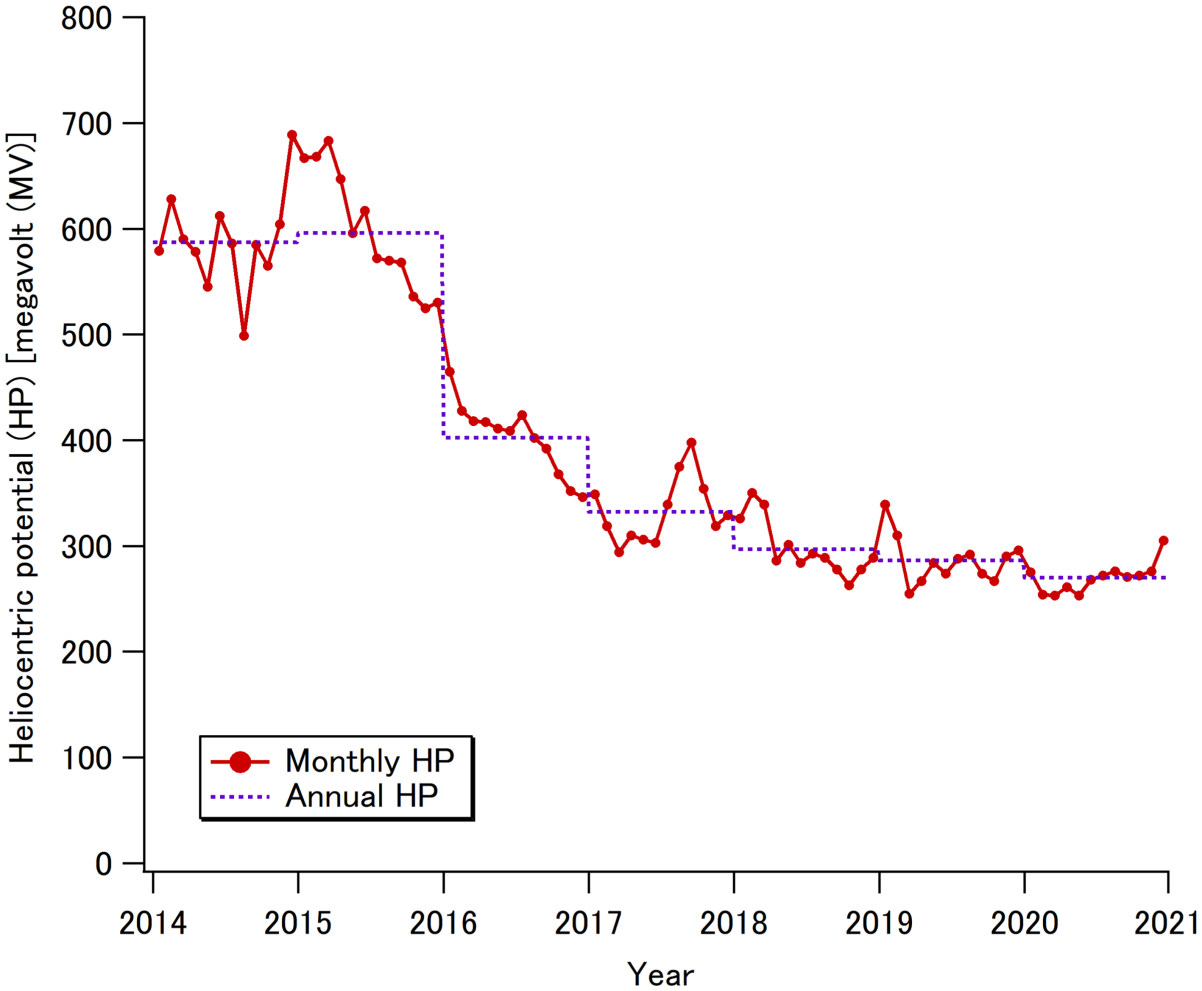
Changes in the monthly and yearly average heliocentric potentials (HPs) over 7 years from January 2014 to December 2020 (21), which were used to calculate the in-flight cosmic radiation doses of flyers in this study.

Solar activity follows a cycle of approximately 11 years, transitioning from minimum to maximum over a period of 5–6 years. Considering this fact and data availability, we focused on the 7-year period from 2014 to 2020, the first year of the COVID-19 pandemic, which covered the transition of solar activity from maximum to minimum. Large solar flares, which could have significantly affected cosmic radiation exposure in aircraft—such as the event observed in January 2005 (20)—did not occur during this period. The monthly and annual changes in solar activity, evaluated using sunspot numbers and referred to as “heliocentric potential (HP),” are shown for the target period in Figure 1 (21).

### Dose calculation for international flights

**Figure 2:**
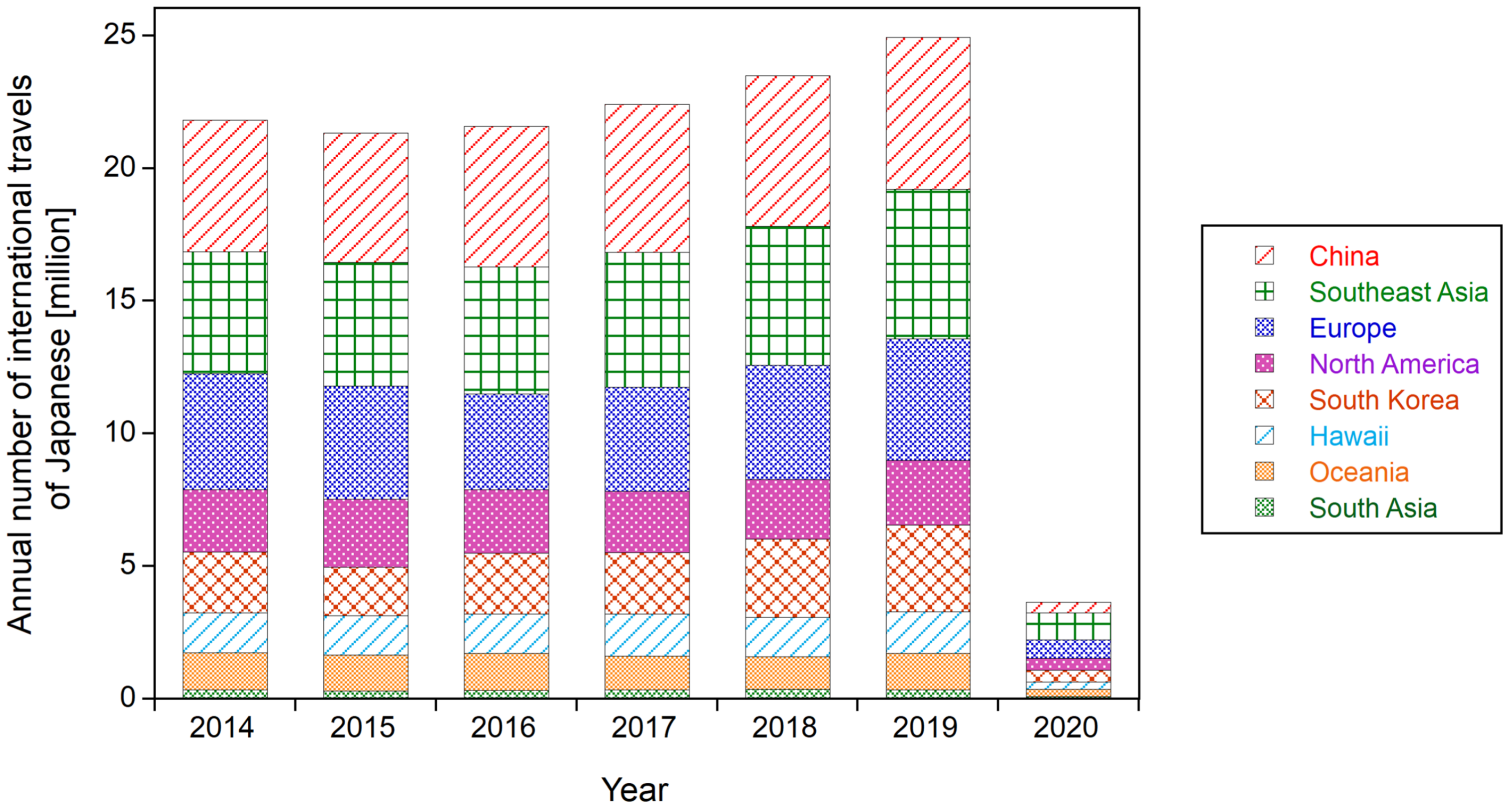
Annual numbers of international air travel by Japanese travelers from 2014 to 2020, classified by eight destination regions (22).

To calculate the effective doses from cosmic radiation onboard aircraft (hereafter referred to as “aviation route doses”) during international flights from Japan, the world, except for Japan, was divided into eight regions. These regions were defined based on the geological differences and the distribution of traveler numbers: South Korea (officially the Republic of Korea), China (including Hong Kong, Taiwan, and Macao), Southeast Asia, South Asia, Oceania, Hawaii, North America, and Europe. The annual number of Japanese travelers to these regions has been officially reported by the Japan Aeronautic Association (JAA) (22), as shown in Figure 2. Since the number of travelers to China in 2020 has not been published (as of November 2024), this figure was estimated based on the number of travelers to Hong Kong in 2020, which was published. The estimate assumed that the ratio (approximately 5%) of Japanese visitors to China and Hong Kong during the period before the COVID-19 pandemic (2014–2019) remained the same in 2020. The total number of international trips to these eight regions accounted for more than 95% of all international flights during the target period. Other regions, such as Africa, the Middle East, and South America, were excluded because the aviation routes to these areas from Japan, including the number of transfers, were unclear. However, the dose contribution from flights to these regions was assumed to be relatively small because of the low number of Japanese travelers and the geomagnetic effects that reduce aviation route doses due to the high cutoff rigidity.

The data shown in Figure 2 reveal that the distribution of air flight destinations remained largely unchanged over this period, including the year of the COVID-19 pandemic (2020). For example, the proportion of Japanese flyers to Europe ranged from 17% (2016) to 20% (2014), with a ratio of 19% in 2020. The proportion of those traveling to North America ranged from 10% in 2018 to 12% in 2020 and those traveling Toto Oceania ranged from 5% in 2018 to 8% in 2020.

**Figure 3:**
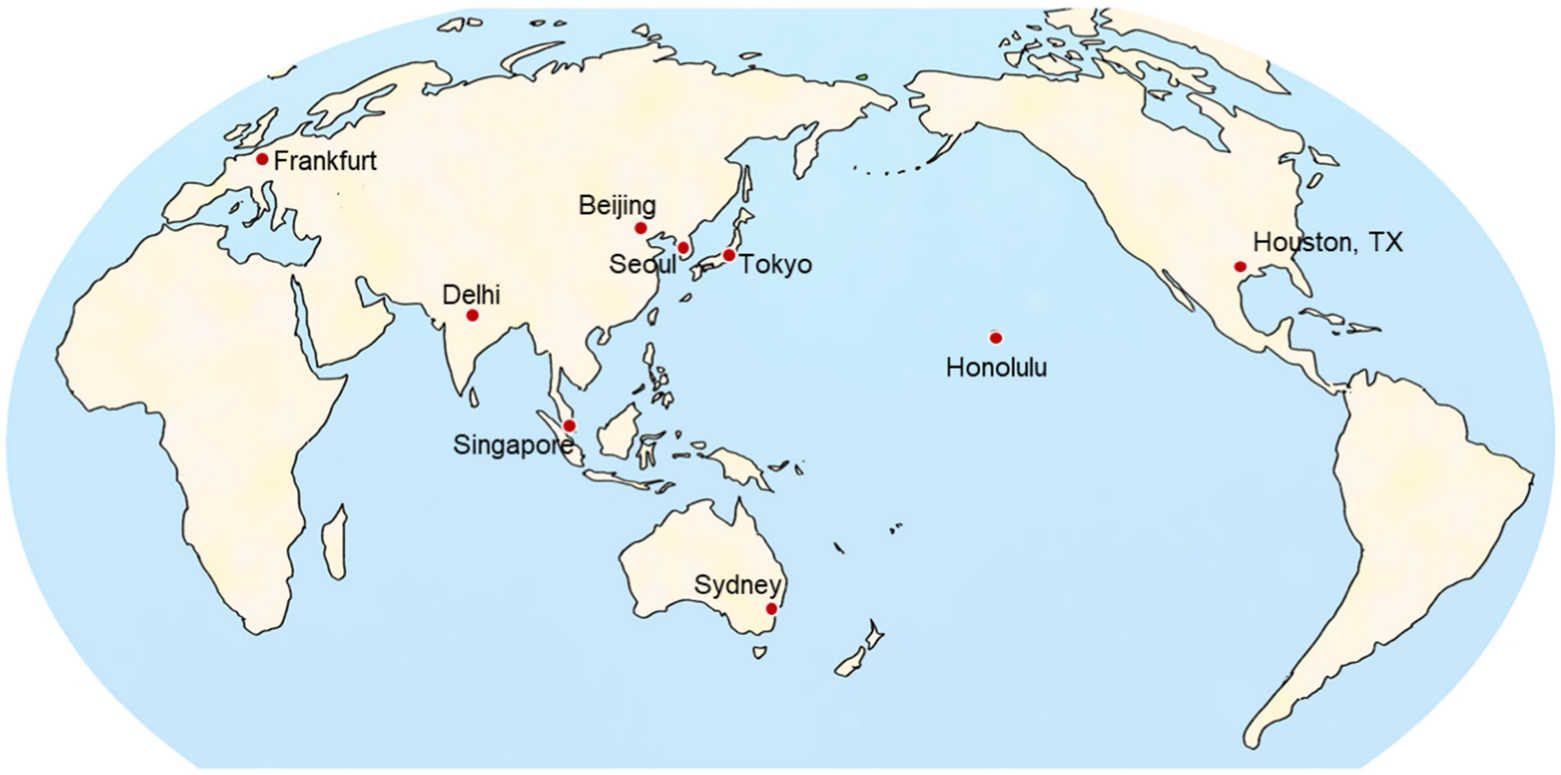
Locations of the cities selected as destinations representing the eight regions used to calculate aviation route doses in international flights from Japan.

The aviation route dose for each region was represented by that for a major city airport that was opened during the pandemic and geographically representative of the region. The selected cities were Seoul for South Korea, Beijing for China, Singapore for Southeast Asia, Delhi for South Asia, Sydney for Oceania, Honolulu for Hawaii, Houston in Texas for North America, and Frankfurt for Europe. The departure and arrival points in Japan was represented by the Tokyo/Haneda Airport. The locations of these cities are shown on the map in Figure 3.

The cosmic radiation dose for each flight was calculated using the program code “JISCARD EX,” which was originally developed by the authors in 2007 for the management of cosmic radiation exposure among aircraft crews working with major airline companies in Japan (10). JISCARD EX is part of the “JISCARD” program package (23), which provides educational information related to cosmic radiation to the public in Japan. The accuracy of the aviation doses calculated using JISCARD EX was validated through comparisons with in-flight measurements (24–27) and other calculation codes developed in other countries (28, 29). For example, the calculated route doses for 68 major commercial flight routes using JISCARD EX and the German code EPCARD. Net matched within ±20% (28), which was considered fully satisfactory for radiological protection purposes. In this study, the most recent radiation and tissue weighting factors provided in the ICRP recommendations (7) were employed to calculate the effective doses.

**Figure 4:**
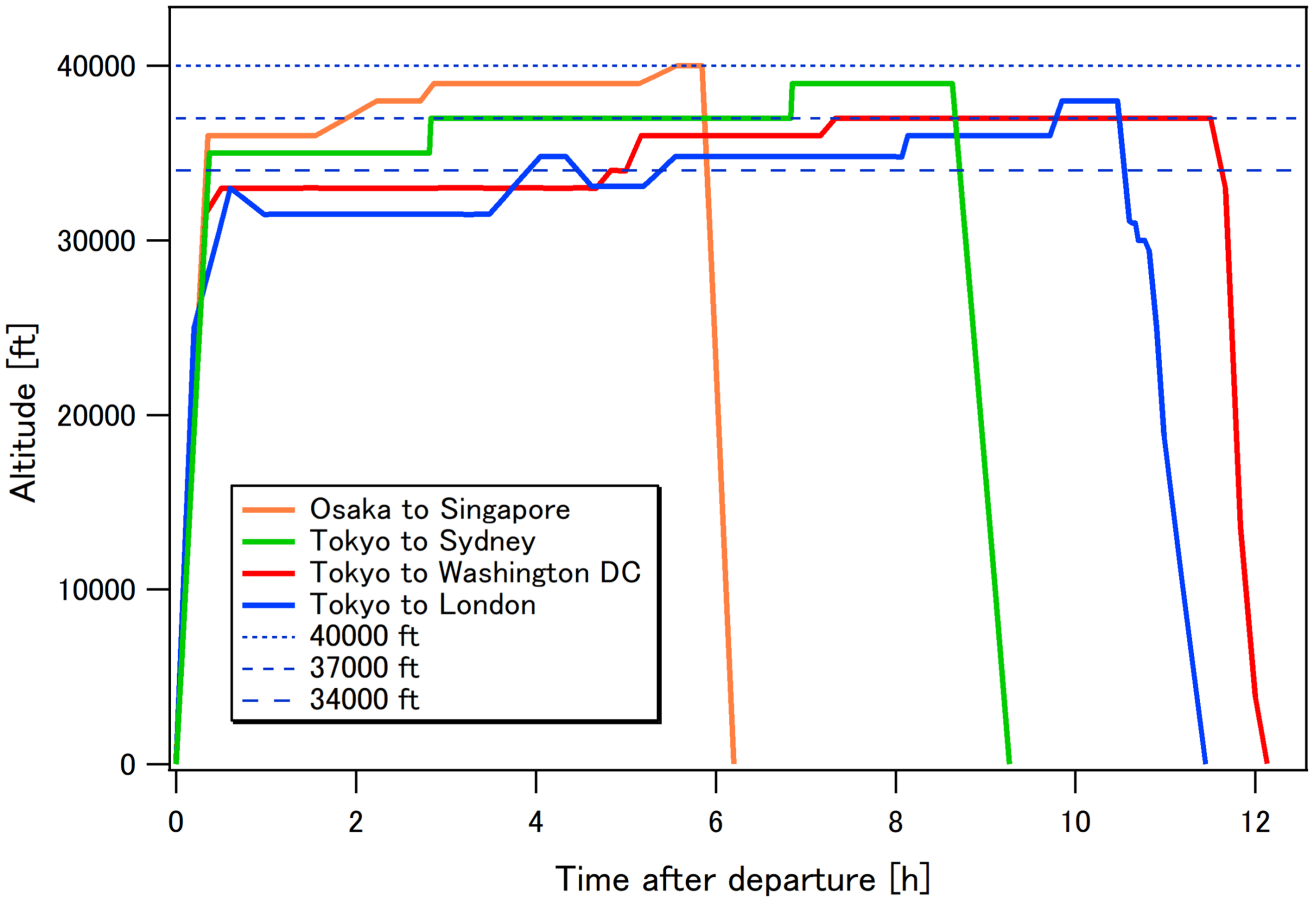
Time changes in the flying altitude of commercial aircraft during four international flights from Japan [reproduced from the data published by Yasuda et al. (26)].

Aviation route doses were calculated under the assumption that all flights followed great-circle routes and adhered to the flight schedules published by major airlines in Japan. Regarding flying altitudes, which could significantly affect dose levels, three cruising altitudes—34,000 ft. (10.4 km), 37,000 ft. (11.3 km), and 40,000 ft. (12.2 km)—were assumed in reference to the flight profiles provided by selected commercial airlines in previous studies (26, 27). For example, the altitude profiles recorded by the authors during four international flights from Japan are shown in Figure 4 (26). The average annual per-capita doses (APCDs) of Japanese flyers and the general population were determined based on the calculated aviation route doses and the number of flyers.

### Dose calculation for domestic flights

**Figure 5:**
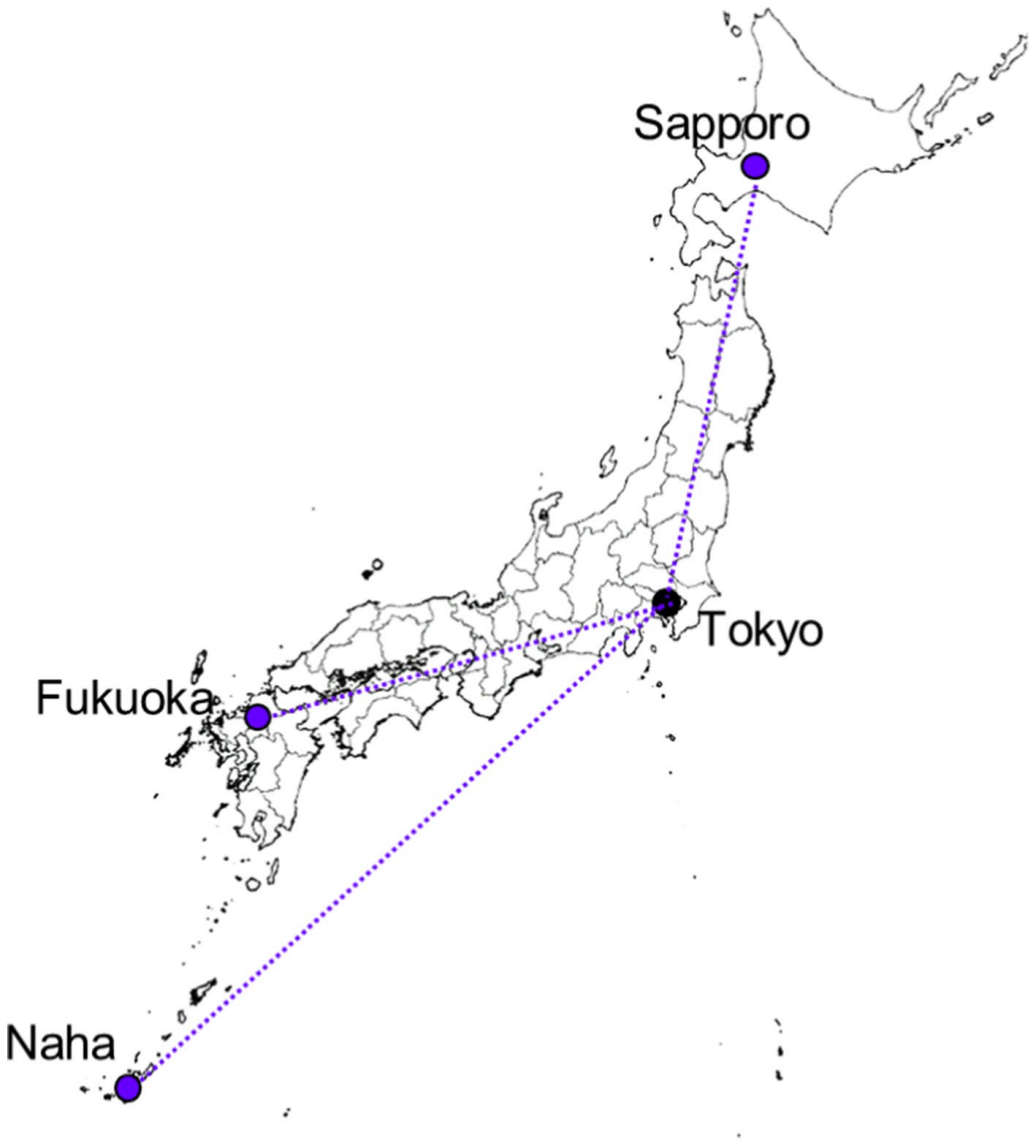
The three flight routes employed for the calculation of aviation route doses from domestic flights in Japan; a unit-distance route dose was determined from these three routes and applied to the calculation of collective doses.

To calculate aviation route doses for domestic flights in Japan, we used a unit-distance route dose for the target period, as performed in a previous study (15). This approach was based on the finding that aviation route doses for domestic flights were primarily dependent on flight time or distance, rather than solar activity or flight route, since they occurred within a limited range of geomagnetic latitudes. In this approach, the collective dose for Japanese travelers was calculated by multiplying the average unit-distance route dose (μSv per 10^3^ km) from three major domestic flights by the annual collective flight distance (man km), as reported by the Japanese governmental body (22). The unit-distance route dose was determined as the average calculated from the three domestic routes with the largest number of flyers in Japan: Tokyo to Sapporo (Hokkaido), Tokyo to Fukuoka (Fukuoka Prefecture), and Tokyo to Naha (Okinawa Prefecture), as shown in Figure 5. The total number of flyers on these three routes accounted for approximately 25% of all domestic flyers during the target period. The aviation route doses were calculated using JISCARD EX (10). In the dose calculation, the cruising altitude was assumed to be 37,000 ft. (11.3 km), and the flying time for each route was determined from the flight time schedule published by major airlines in Japan.

## Results

### Doses from international flights

**Figure 6:**
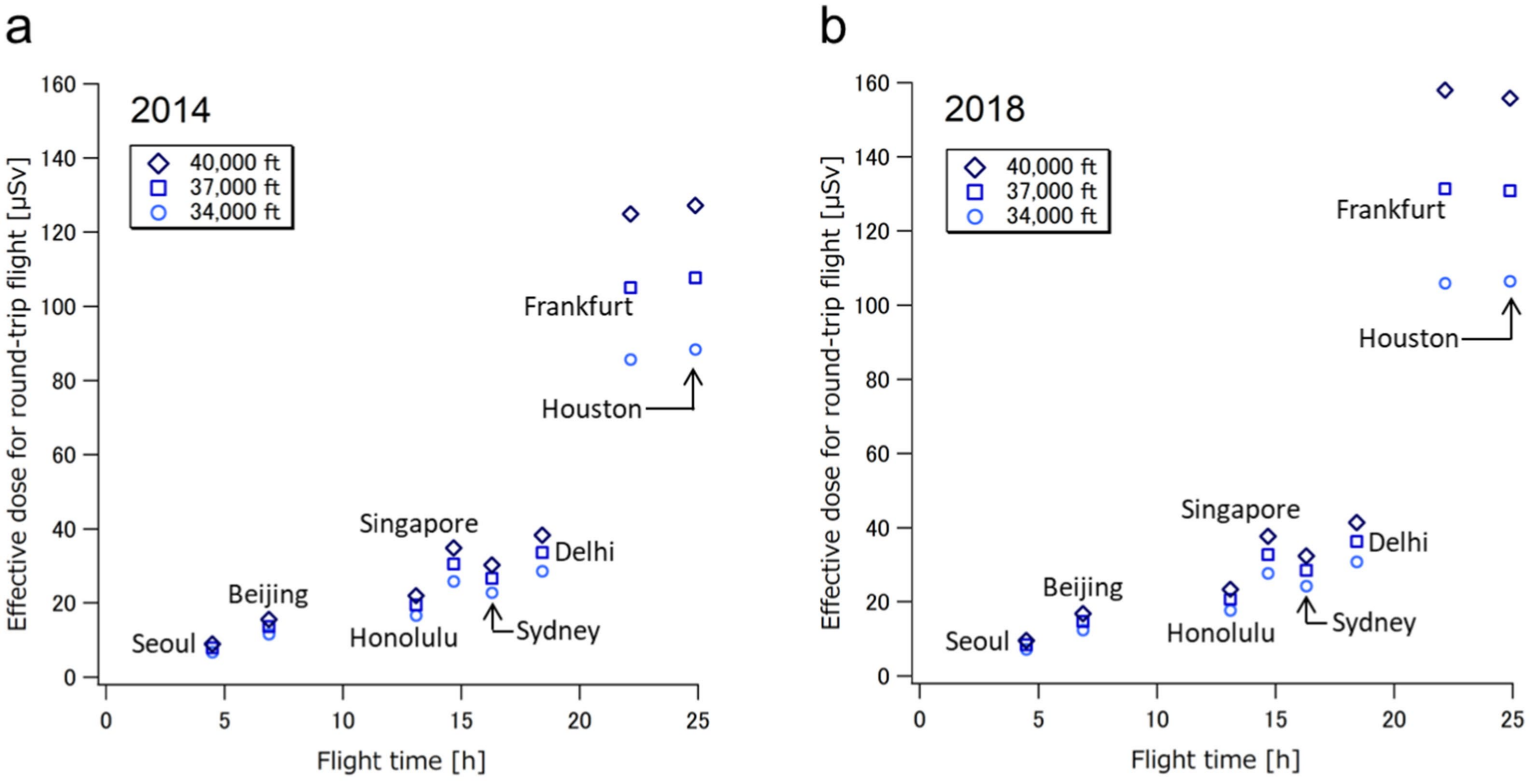
Calculated aviation route doses for round-trip flights to the cities representing seven out of the eights regions (Figure 3) at three cruising altitudes (34,000, 37,000, and 40,000 ft) as a function of flight time for 2014 **(a)** and 2018 **(b)**.

In Figure 6, the calculated cosmic radiation doses for round-trip flights to cities representing seven of the eight regions (Figure 3) at three cruising altitudes (34,000, 37,000, and 40,000 ft) are plotted as a function of flight time for the years 2014 and 2018. The aviation route doses for flights to Europe and North America were notably higher than those for flights to Asia and Oceania because the former flights have longer durations and take higher-latitude routes near the North Pole, while the latter flights have shorter durations and lower-latitude routes. In addition, the effects of the cruising altitudes were clearly observed only in the flights to Europe and North America. These results are attributed to the distribution of Earth’s geomagnetic cutoff rigidity, which is lowest over the polar region and highest over the equator (5). Compared to the aviation doses in 2014 and 2018, the calculated doses for relatively short flights to Asia and Oceania were nearly the same, indicating that these low-latitude flights are minimally affected by changes in solar activity, which follows an 11-year cycle. The dose levels showed a tendency to gradually increase with flying time.

**Figure 7:**
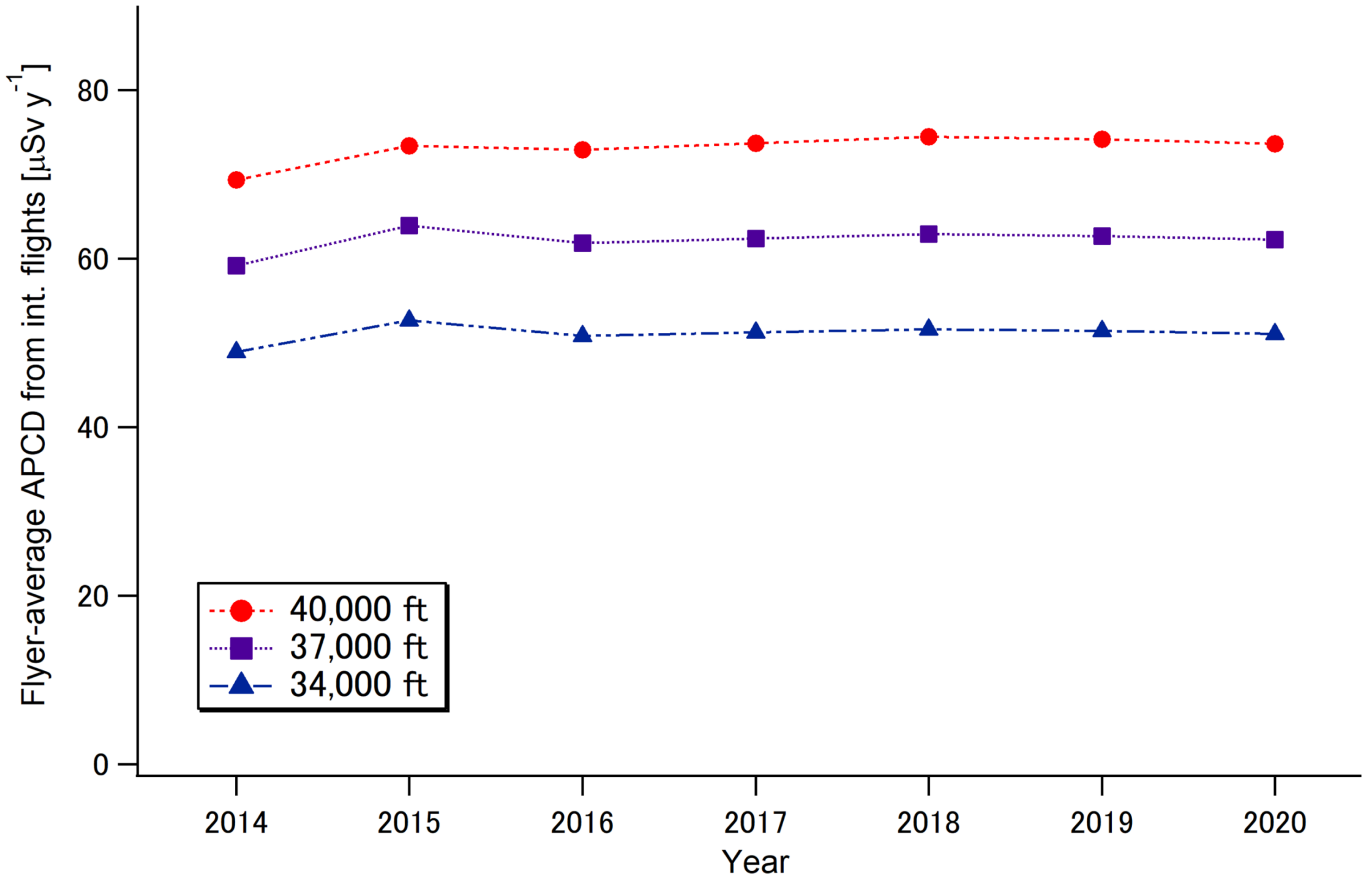
Calculated flyer-average per-capita doses (APCDs) for Japanese flyers from cosmic radiation during international flights for the period 2014–2020.

The annual number of Japanese flyers (30), the calculated annual collective effective dose (the sum of route doses multiplied by the number of Japanese flyers) for international flights, and the flyer-average annual per-capita dose (APCD) (the quotient of the collective dose divided by the number of Japanese travelers) from international flights over the target period of 2014–2020 are summarized in Table 1, and the calculated flyer-average APCDs are plotted as a function of year in Figure 7. The flyer-average APCD for the Japanese flyers was estimated to be 59–63 μSv y^−1^, which is notably higher than the cosmic radiation level encountered on the ground by the general public (approximately 1 μSv per day) (6). The flyer-average APCD changed little even during the pandemic year (2020), whereas the collective dose decreased markedly to approximately 15% of the pre-pandemic level in 2020, reflecting the significant reduction in overseas travel (Figure 2). It should be noted that the number of trips per flyer decreased from 1.24–1.32 in the pre-pandemic period (2014–2019) to 1.15 in 2020.

**Table 1:** The number of Japanese international flyers, annual number of trips per international flyer, annual collective effective doses from international flights, and flyer-average annual per-capita doses (APCDs) from international flights during the period of 2014–2020.

Year	Annual number of Japanese international flyers	Annual number of trips per international flyer	Annual collective effective dose from international flights (man Sv)*	Flyer-average APCD from international flights (μSv y^−1^)*
2014	16,903,000	1.29	1,000 (827–1,173)	59.2 (48.9–69.4)
2015	16,213,766	1.32	1,036 (854–1,190)	63.9 (52.7–73.4)
2016	17,116,420	1.26	1,058 (870–1,248)	61.8 (50.8–72.9)
2017	17,889,292	1.25	1,116 (917–1,319)	62.4 (51.2–73.7)
2018	18,954,031	1.24	1,193 (978–1,411)	62.9 (51.6–74.5)
2019	20,080,669	1.24	1,259 (1,032–1,489)	62.7 (51.4–74.2)
2020	3,174,219	1.15	198 (162–234)	62.3 (51.1–73.6)

*The dose value calculated at a cruising altitude of 37,000 ft, followed by those for altitudes between 34,000 and 40,000 ft in parentheses.

### Doses from domestic flights

Table 2 shows the annual number of domestic flyers in Japan, collective flight distance, collective effective dose (the product of the collective flight distance and the unit distance dose), and the derived flyer-average APCD (the quotient of the collective dose divided by the number of Japanese travelers) from domestic flights during the period 2014–2020. As observed in the results for international flights (Table 1), the flyer-average APCD from domestic flights in Japan was not affected by the pandemic (1.82–1.86 μSv y^−1^ in 2014–2019; 1.87 μSv y^−1^ in 2020), whereas the collective dose significantly decreased in 2020 to one-third of the pre-pandemic level. This APCD level was equivalent to the dose encountered during a domestic flight of approximately 900 km, which is nearly the same distance as that from Tokyo to Sapporo (Figure 5).

**Table 2:** The number of domestic flyers in Japan, annual collective distance of domestic flights, collective effective doses from domestic flights, and flyer-average annual per-capita doses (APCDs) from domestic flights in Japan during the period of 2014–2020.

Year	Number of domestic flyers in Japan	Annual collective distance of domestic flights (10^3^ man km)	Collective effective dose (10^3^ man Sv)	Flyer-average APCD from domestic flights (μSv y^−1^)
2014	94,504,500	86,148,872	172.3	1.82
2015	95,874,432	87,913,827	175.8	1.83
2016	97,203,255	89,589,278	179.2	1.84
2017	102,119,109	94,427,161	188.9	1.85
2018	103,902,583	96,170,616	192.3	1.85
2019	101,872,143	94,488,458	190.0	1.86
2020	33,767,529	31,543,287	63.09	1.87

### Population average dose

**Figure 8:**
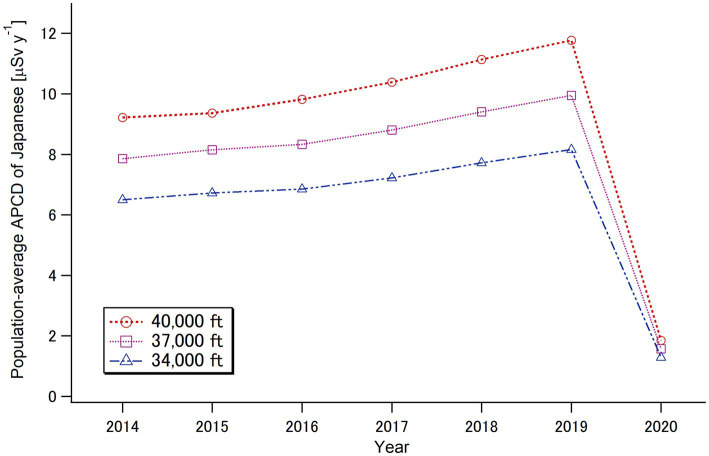
Calculated annual per-capita doses (APCDs) as the Japanese population average from cosmic radiation during air travels for the period of 2014–2020.

Based on Japanese population data (31) and the calculated collective doses from international and domestic flights (Tables 1, 2), population-average APCDs for Japanese individuals from in-flight cosmic radiation exposure during the target period of 2014–2020 were calculated, as shown in Table 3 and Figure 8. Since the number of Japanese flyers who took international flights represented a small proportion of the Japanese population, the population-average APCD from international flights was significantly smaller than the flyer-average APCD (Table 1). However, the population-average dose from domestic flights was approximately 70% of the flyer-average APCD, indicating that a large number of Japanese people use aircraft for domestic transportation. The population-average APCD for the Japanese population significantly decreased in 2020 due to the COVID-19 pandemic, dropping to <20% of the 2019 level.

**Table 3:** Number of the Japanese population, population-average annual per-capita doses (APCDs) from international flights, APCDs from domestic flights, and population-average APCDs of the Japanese population during the period of 2014–2020.

Year	Number of the Japanese population (10^3^)	APCD from international flights (μSv y^−1^)*	APCD from domestic flights (μSv y^−1^)	Population-average APCD of the Japanese population (μSv y^−1^)*
2014	127,237	7.86 (6.50–9.22)	1.35	9.21 (7.85–10.57)
2015	127,095	8.15 (6.72–9.36)	1.38	9.53 (8.10–10.74)
2016	127,042	8.33 (6.85–9.82)	1.41	9.74 (8.26–11.23)
2017	126,919	8.80 (7.22–10.39)	1.49	10.29 (8.71–11.88)
2018	126,749	9.41 (7.72–11.13)	1.52	10.93 (9.24–12.65)
2019	126,555	9.95 (8.16–11.77)	1.49	11.44 (9.65–13.26)
2020	126,146	1.57 (1.29–1.85)	0.50	2.07 (1.79–2.35)

*The dose value calculated at a cruising altitude of 37,000 ft, followed by those for altitudes between 34,000 and 40,000 ft in parentheses.

## Discussion

In this study, we present the first quantitative estimates of cosmic radiation exposures for commercial flight passengers (flyers) during the period of 2014–2020, which included the COVID-19 pandemic, using official records provided by governmental bodies in Japan. Accordingly, the flyer-average annual per-capita doses (APCDs) from international flights at a typical cruising altitude of 37,000 ft. (11.3 km) were approximately 60 μSv y^−1^, while the APCDs from domestic flights were approximately 2 μSv y^−1^ over the target period. Interestingly, the flyer-average APCD was not affected by the COVID-19 pandemic. The observed stability in the flyer-average APCD, regardless of changes in solar activity (Figure 1), can be attributed to the fact that the relative change in the number of flyers during the pre-pandemic period (2014–2019) occurred mainly on low-latitude flights in Asia, such as in China, Southeast Asia, and South Korea, which were considered unaffected by changes in solar activity. During the pandemic period (2020), although the relative contribution of low-latitude flights slightly decreased, this effect was balanced by the reduction in the number of trips per international flyer (Table 1). Meanwhile, the population-average APCD notably decreased by more than 80% in 2020 compared to the pre-pandemic period (2014–2019) population, reflecting the significant reduction in the number of Japanese travelers during the pandemic period.

These annual dose levels from air travel are negligible compared to the APCD from all-natural radiation sources, which averages approximately 2 mSv for the Japanese population (32). However, aviation doses can vary among individuals; some frequent flyers may have received relatively high doses >1 mSv, as reported for aircraft crews (5). While it is currently difficult to collect personal data from accessible travel records, future studies should aim to assess the individual doses for frequent flyers from in-flight cosmic radiation. It should be noted that we attempted to calculate APCDs for the years 2021–2023 after the COVID-19 pandemic but found that data on the number of Japanese travelers to several major countries, including China and France, were still unavailable. In addition, flight routes between Japan and Europe have changed significantly due to the Russia–Ukraine War, which began in February 2022. As detailed records of these route changes were not made publicly available, we determined that reliable estimates of APCDs for Japanese flyers for 2022 or later would be difficult to obtain. Nevertheless, since the number of Japanese travelers leaving the country in 2021 and 2022 was officially reported to be 512,244 and 2,771,770 (30), respectively, it was presumed that APCDs for Japanese flyers from international flights must have been significantly lower in recent years than in 2020, as well as in the pre-pandemic period. In particular, as the number in 2021 (5.12 × 10^5^) was only one-sixth of the number in 2020 (3.17 × 10^6^), the population-average APCD in 2021 was presumed to have been markedly lower.

**Figure 9:**
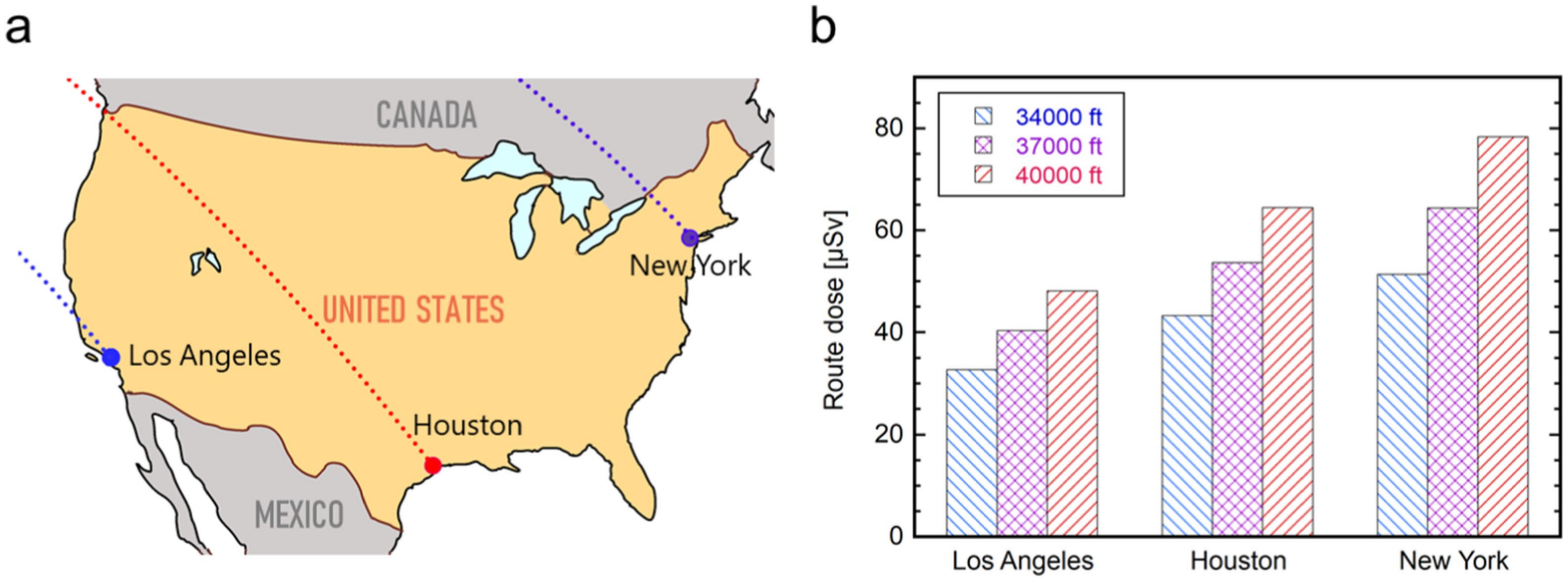
**(a)** Locations of the three cities in the United States (Los Angeles, Houston, and New York) and **(b)** calculated route doses from Tokyo to the three cities when flying in November 2020 at constant cruising altitudes of 34,000, 37,000, and 40,000 ft. The dotted lines in the map **(a)** indicate great circle routes from Tokyo.

This study has some limitations. First, the accurate number of Japanese travelers who took international flights could be higher than the figures covered in this study (Table 1) as flights to some regions, such as Africa, the Middle East, and South America, were excluded due to a lack of information on exact flight routes. Second, some uncertainty was introduced by the simplified procedures in the route dose calculation, such as dividing the world into eight regions and selecting only one representative city for each region. In particular, the potential uncertainty resulting from the simplification of flights to North America is considered significant because of the larger regional size and lower geomagnetic latitude (i.e., higher cosmic radiation dose rate). Considering this uncertainty, we calculated aviation route doses for flights from Tokyo to three distant cities in the United States (Los Angeles, Houston, and New York), assuming great circle routes at constant cruising altitudes of 34,000 ft., 37,000 ft., and 40,000 ft., as shown in Figure 9. Accordingly, we determined that Houston could appropriately be chosen as a focal-point airport in North America for Japanese travelers because the route dose for Houston fell between that for Los Angeles on the west coast and that for New York on the east coast. However, the potential uncertainty due to this simplification should be investigated in more comprehensive ways. In addition, possible air travel subsequently made from the first destination country could not be considered, despite the speculation that a considerable number of travelers flew somewhere to or from other countries. Moreover, it is necessary to improve the accuracy of the model for calculating cosmic radiation dose rates in the atmosphere based on recent observations at aviation altitudes (33, 34). More precise dose calculations for flyers would need to consider the spatial distribution of dose rates in a cabin area, which could be affected by the complex nuclear interactions of cosmic radiation particles with the aircraft body, fuel, onboard instruments, luggage, and passengers, as observed in previous in-flight measurements (35, 36). Further efforts to reduce these potential uncertainties are needed to achieve a more reliable dose assessment.

## Conclusion

This is the first study to present recent trends in cosmic radiation exposure of the general public onboard commercial aircraft, focusing on the impact of the COVID-19 pandemic on the in-flight doses of Japanese passengers (flyers). As expected, due to the reduction in the number of flyers, the population-average annual per-capita dose (APCD) of Japanese flyers notably decreased in the first year of the pandemic (2020), whereas the flyer-average APCD changed little. These findings are expected to contribute to ongoing discussions on the overall impact of the COVID-19 pandemic on public health by clarifying the changes in radiological risk due to travel restrictions. In addition, the quantitative data presented in this study will be useful for the members of the public to deepen their understanding of varying situations of naturally occurring radiation exposure as a baseline for radiological health risk and to assess what level of additional exposure should be considered significantly harmful. Given the uncertainties in this study due to the simplified procedures and missing detailed information, we plan to conduct more comprehensive research in collaboration with airlines to improve the accuracy and reliability of our findings, covering the subsequent pandemic period (2021–present).

## Data Availability

The raw data supporting the conclusions of this article will be made available by the authors, without undue reservation.
